# Seasonal sea ice cover as principal driver of spatial and temporal variation in depth extension and annual production of kelp in Greenland

**DOI:** 10.1111/j.1365-2486.2012.02765.x

**Published:** 2012-07-26

**Authors:** Dorte Krause-Jensen, Núria Marbà, Birgit Olesen, Mikael K Sejr, Peter Bondo Christensen, João Rodrigues, Paul E Renaud, Thorsten JS Balsby, Søren Rysgaard

**Affiliations:** * Department of Bioscience, Aarhus UniversityVejlsøvej 25, DK-8600, Silkeborg, Denmark; † Department of Global Change Research, IMEDEA (CSIC-UIB), Institut Mediterrani d'Estudis AvançatsMiquel Marquès 21, 07190, Esporles (Illes Balears), Spain; ‡ Department of Applied Mathematics and Theoretical Physics, Centre for Mathematical SciencesWilberforce Road, Cambridge, CB3 0WA, United Kingdom; § Fram Centre for Climate and the Environment, Akvaplan-nivaN-9296, Tromsø, Norway; ¶ Greenland Climate Research Centre (Co. Greenland Institute of Natural Resources)Kivioq 2, Box 570, 3900, Nuuk, Greenland; ‖ Centre for Earth Observation Science, CHR Faculty of Environment Earth and Resources, University of Manitoba499 Wallace Building, Winnipeg, MB R3T 2N2, Canada; ** Arctic Research Center, Aarhus UniversityDK-8000, Århus C, Denmark; †† Department of Bioscience, Aarhus UniversityOle Worms Allé, Building 1135, DK-8000, Århus C, Denmark

**Keywords:** Arctic, climate change, depth limit, kelp, production, sea ice cover

## Abstract

We studied the depth distribution and production of kelp along the Greenland coast spanning Arctic to sub-Arctic conditions from 78 °N to 64 °N. This covers a wide range of sea ice conditions and water temperatures, with those presently realized in the south likely to move northwards in a warmer future. Kelp forests occurred along the entire latitudinal range, and their depth extension and production increased southwards presumably in response to longer annual ice-free periods and higher water temperature. The depth limit of 10% kelp cover was 9–14 m at the northernmost sites (77–78 °N) with only 94–133 ice-free days per year, but extended to depths of 21–33 m further south (73 °N–64 °N) where >160 days per year were ice-free, and annual production of *Saccharina longicruris* and *S. latissima,* measured as the size of the annual blade, ranged up to sevenfold among sites. The duration of the open-water period, which integrates light and temperature conditions on an annual basis, was the best predictor (relative to summer water temperature) of kelp production along the latitude gradient, explaining up to 92% of the variation in depth extension and 80% of the variation in kelp production. In a decadal time series from a high Arctic site (74 °N), inter-annual variation in sea ice cover also explained a major part (up to 47%) of the variation in kelp production. Both spatial and temporal data sets thereby support the prediction that northern kelps will play a larger role in the coastal marine ecosystem in a warmer future as the length of the open-water period increases. As kelps increase carbon-flow and habitat diversity, an expansion of kelp forests may exert cascading effects on the coastal Arctic ecosystem.

## Introduction

The Arctic is the fastest warming region of the globe and temperatures are projected to increase during the 21st century at twice the global average rate (IPCC, 2007). One of the most significant impacts of increasing temperature in the Arctic is the marked reduction in sea ice cover, which is projected to result in an ice-free Arctic Ocean in summer within the next few decades (Wang & Overland, 2009). Reduced sea ice extent is expected, in turn, to trigger physical, chemical and biological changes with potentially large impacts on the Arctic marine ecosystem (Duarte *et al*., 2012). To date, there is only limited evidence on how marine biota responds to the rapid Arctic warming and the retreat of sea ice (Wassmann *et al*., 2011). Time series, which allow retrospective analyses of responses to climate change, are scarce, and this limits the possibilities of quantifying how the marine ecosystem responds to different climate forcing. However, ecological studies of ecosystem structure along latitudinal gradients in climatic conditions may provide an additional means to anticipate responses to warming.

Benthic primary producers are important contributors to total primary production of shallow coastal waters where light reaches the sea floor (Gattuso *et al*., 2006; Glud *et al*., 2009), as opposed to oceanic systems where phytoplankton is the only primary producer. Kelp, large perennial brown algae in the order Laminariales, grow even in cold high-Arctic ecosystems where long polar winters and extensive sea ice cover confine the productive season with light to a short period in summer (Dunton, 1985; Borum *et al*., 2002). Despite the conditions, the primary production of kelps and other benthic macroalgae can contribute over 20% of the total primary production (dominated by phytoplankton and at much less extent, sea ice algae and benthic microalgae) in a near-shore high-Arctic area, and even exceed pelagic primary production at water depths shallower than 20 m (Glud & Rysgaard, 2007; Krause-Jensen *et al*., 2007). This primary production enters the food web through grazing, exudation of dissolved organic carbon and as detritus (Mann, 1973; Duggins *et al*., 1989). In addition, kelp habitats provide substrate for sessile animals, shelter from predation, and protection against wave action and currents. Kelps are therefore key species in coastal ecosystems affecting carbon flow as well as biodiversity (Dayton, 1985; Bruno & Bertness, 2001; Lippert *et al*., 2001; Steneck *et al*., 2002). Despite their ecological importance, only very limited information exists on the *in situ* growth, abundance and depth extension of kelp forests along the Greenland coast and the physical factors responsible.

The availability of light for fuelling marine primary production varies markedly along the Greenland coast from the high-Arctic north to the sub-Arctic south due to differences in the extent and duration of sea ice cover and latitudinal differences in the length of the dark period, in addition to local differences in weather conditions and light attenuation. The ice-free period increases from only about 2–3 months in northern Greenland to ice-free conditions during almost the entire year in the south (Sejr *et al*., 2009). Pelagic primary production in near-shore waters has been found to increase along such spatial gradients of increased duration of the open-water period (Rysgaard & Glud, 2007). Time series of net primary production of phytoplankton of the Arctic Ocean also indicate a 20% increase from 1998 to 2010 in response to larger and longer open-water periods, as evaluated from satellite images (Arrigo & van, 2011), and model simulations predict increases in pelagic primary production in areas where nutrient supply supports growth (Slagstad *et al*., 2011). Increased production of sea-urchins and mussels southwards along the Greenland coast also suggests that the availability of food increases southwards (Blicher *et al*., 2007; Sejr *et al*., 2009). In line with these findings, southward increases in production and depth extension can be expected for benthic Arctic macroalgae, most of which are of north-Atlantic or Pacific origin (Lüning, 1990; Wilce, 1996; Wulff *et al*., 2009) with temperature optima for growth above ambient temperatures (Bischoff & Wiencke, 1993; Müller *et al*., 2009).

This study aimed to (1) quantify the depth distribution and annual growth of kelps along the Greenland coast from the high-Arctic with pronounced seasonal sea ice cover, long dark periods and low temperatures to the sub-Arctic with less sea ice and higher temperatures, (2) quantify the growth response of high-Arctic kelp to annual changes in sea ice cover and (3) predict the response of Greenland's kelp to global warming based on the observed latitudinal and temporal coupling of kelp depth distribution and production to climate forcing.

## Materials and methods

### Study sites

**Fig. 1:**
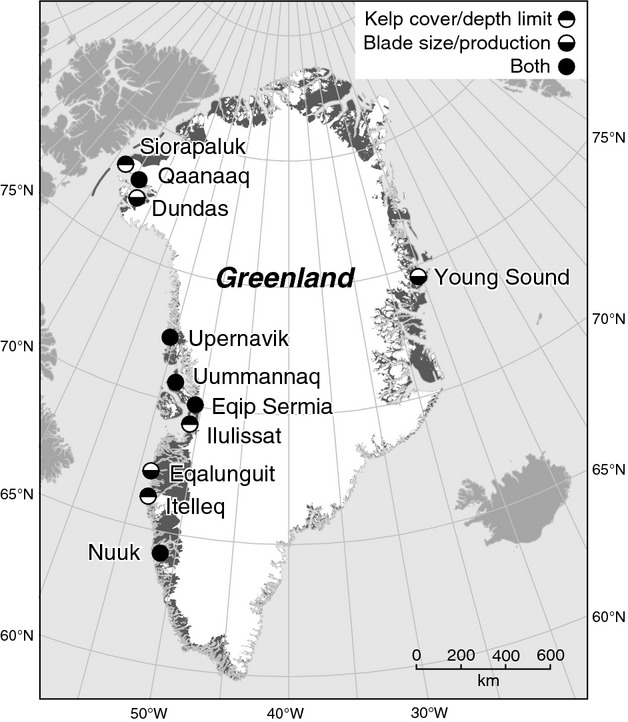
Map of sites along the Greenland coast with information from video surveys of the depth extension of the kelp belt (top of circle filled) and/or on production, size structure, nutrient content and reproductive status of kelp (*S. latissima* and/or *S. longicruris*) (bottom of circle filled).

We studied the kelp forest along the Greenland coast over a gradient from high-Arctic Siorapaluk at 78 °N in northwestern Greenland and Young Sound at 74 °N in northeastern Greenland to sub-Arctic Nuuk at 64 °N in southwestern Greenland (Fig. 1). The majority of the sites along Greenland's west coast from Siorapaluk at 78 °N to Itelleq at 67 °N were studied in the period 27 August to 10 September 2009 during a cruise with the vessel MS FRAM, operated by Hurtigruten AS, Norway. An additional site (Upernavik 73 °N) was sampled during a similar cruise by MS FRAM in August 2010, and a site in the western part of the Disko Bay (Eqalunguit 67 °N) was sampled in September 2009. Moreover, the study includes data collected in conjunction with the Greenland Ecosystem Monitoring (GEM, http://www.dmu.dk/greenland/klimaeffekter/gem/) at Nuuk in August 2008–2009 and at Young Sound in August 1999 (Borum *et al*., 2002) and 2003–2011. The data thus describe a wide spatial range of climatic conditions supplemented with a 13-year time series from northeastern Greenland and all in all represents a large section of Greenland's almost 90 000 km long coastline (measured by M. Stjernholm, Aarhus University, based on 1:250 000 topographic maps, provided by the Geological Survey of Denmark and Greenland and the National Survey and Cadastre, Denmark). The investigated sites were relatively protected, situated in bays or between islands, representing coastal slopes of ca. 1–26 °, and the seafloor varied from sandy with scattered boulders and stones to rocks (Table S1).

### Sea ice cover, light climate and water temperature

The variation in sea ice conditions along Greenland's west coast was characterized on the basis of sea ice concentration data in 2009 obtained from passive microwave imagery processed with the Enhanced NASA Team algorithm (Markus & Cavalieri, 2000) and archived and distributed by the National Snow and Ice Data Center (Cavalieri *et al*., 2004). The length of the ice-free period was calculated with an algorithm described by Rodrigues (2009). Information on sea ice cover was obtained from all sites except Eqip Sermia where the position studied was too surrounded by land to return a useful value for the sea ice cover. Information on sea ice cover in Young Sound was based on daily photos revealing the time of sea ice formation and break-up as part of the GEM. The seasonal period of sea ice cover was overlain with information on the astronomical dark period (http://gandraxa.com/length_of_day.xml) to estimate the duration of the open-water period with light at each of the sampling sites. This period was quantified as the number of days in which there was no ice and some light. We chose ‘days’ as *x*-axis unit rather than a more detailed account of the light period in hours to highlight the increasingly uneven seasonal distribution of light northwards and the corresponding increased need for the kelp to cope with a long dark period.

Light attenuation in the water column was obtained through one-time measurements of the diffuse attenuation coefficient (K_d_) using an underwater cosine PAR sensor (Licor, type LI-192SA) at the sites visited during the FRAM cruise in August 2010. Light was measured subsurface, at 1 metre intervals until 10 m depth and then at 2 m intervals. Light attenuation at Nuuk and Young Sound was obtained from the GEM.

Water temperature along Greenland's west coast from Qaanaaq to Itelleq was measured from the FRAM vessel at 5 m below the sea surface during the cruise in August/September 2009. These one-time measurements are coarse and only useful for indicating large-scale differences. In addition, temperature was recorded every 30 min by HOBO loggers (HOBO Pendant® Temperature/Light Data Logger; Onset Computer Corporation, Bourne, MA, USA) deployed for 1 year at 10 m depth at the northernmost sampling sites on Greenland's west and east coasts (Siorapaluk; Young Sound) as well as from August 2009 to May 2010 at Nuuk. August data from these loggers were used to characterize the temperature near the kelp sampling sites at Young Sound and Nuuk. The loggers were attached to vertical lines held in place by a subsurface buoy and a double-anchor. For the time series analyses (1999–2011), water temperatures from Young Sound were extracted from the GEM database as mean August values for the depth range 0–45 m (Jensen & Rasch, in press).

### Depth extension of the kelp forest

Cover and depth limits of the kelp forest were recorded by underwater video (sport QX, 560 TVL 0.1 Lux, “http://www.LH-camera.dk”) during the FRAM cruises at seven sites along the west coast (Fig. 1). The camera was held by a tow-fish at an angle of about 45˚ and connected to a 50 m cable, which was carefully lowered from a zodiac to about one metre above the sea floor and towed from the shore to the deepest occurrence of erect macroalgae. Recordings were shown directly on a screen on the zodiac, and logged as ‘.avi’ files. The camera had a built-in GPS and time logger and a built-in depth sensor which, however, was not working during the 2009 cruise, during which the echo sounder of the zodiac was used for depth recordings. The depth at the time of sampling was corrected for tides and expressed relative to the average annual tidal level using tidal information from the nearest harbour (http://frv.dk/Maalinger/Farvandsmaalinger/Documents/tidevand/Tide10%20GR.pdf) (Richter *et al*., 2011). Additional video recordings of total macroalgal cover from August 2008 in the outer part of Kobbefjord, Nuuk, where kelp dominated the macroalgal community (Jensen & Rasch, 2009) were also included. Two to seven transect lines/depth gradients, the number depending on logistics, were recorded at each site. Each video recording was analysed as point observations (few seconds of video) of the percent cover of kelp relative to the seafloor at specific depths and supplemented with comments on species composition. The number of point observations (18–95) along a transect line depended on the depth extension of the kelps and the slope of the coastline. On the basis of video information, we identified the maximum depth of 50%, 10% and 1% algal cover for every transect line and calculated the mean for each site.

### Growth performance of *Saccharina latissima* and *S. longicruris*

*Saccharina latissima* and *S. longicruris* were sampled for comparative studies of growth, size structure, reproduction and tissue nutrient content along the Greenland coast as they have a large geographical distribution range (Lüning, 1990; Müller *et al*., 2009) and generally dominated the kelp forests along the entire latitude range studied (Table S1). The two species have quite similar morphology and were once considered conspecific by some scientists (Chapman, 1974), even though *S. longicruris* has a longer and hollow stipe. These kelps have a perennial stipe, produce new blade tissue every year and were found to have similar growth rates at a site where both occurred (Egan & Yarish, 1990). The new blade is initiated in winter at the intersection between the stipe and the existing blade, and new tissue pushes older tissue towards the tip where it gradually wears off. The blade exhibits maximum growth (in length) in spring and early summer and completes the annual length growth in late summer (Borum *et al*., 2002). In northeastern Greenland, the old blade of *S. latissima* may remain for one or two years, clearly separated from younger blades by constrictions, making it possible to accurately estimate annual growth based on the size of the annual blade sampled in late summer (Lund, 1951; Dunton, 1985; Borum *et al*., 2002). On the west coast, the kelp only rarely maintained the previous year's blade and the constriction between old and new tissue; however, tapering of blades towards the tip indicated that they were generally almost complete, and that the length of the blade therefore provided a minimum estimate of annual growth (Jensen & Rasch, 2009). Further south, in the temperate zone, blades of *Saccharina* may turn over several times in a year and measurements of annual growth there require repeated marking of the blades (e.g. Mann, 1973).

The kelp, *S. longicruris* and/or *S. latissima*, depending on the harvest, was sampled from a water depth of 10 m, slightly deeper at Uummannaq (∼13 m) and shallower at Nuuk (∼6 m). A total of eight sites were sampled (Fig. 1), four representing each species. Sampling was conducted from a zodiac by dragging a metal plant rake with spikes emerging from a central basal disc, along the seafloor, except at Nuuk and Equalunguit where a diver collected the kelp. The rake may tend to sample larger individuals, but as this is equal for all sites and smaller individuals were only used to illustrate allometric relationships, it should not create a bias. At Nuuk sampling has, over the years, been conducted by diver as well as by rake without any apparent difference in the mean size of mature specimens (Jensen & Rasch, in press). The specimens were carefully retrieved, and length and width of the stipe and the new blade (at ¼, ½ and ¾ of the length), length of the fertile sporulated blade area as well as biomass of stipe and blade were measured. A total of 24–64 specimens were sampled at each site except at Eqalunguit and Young Sound, where samplings in 2009 included measurements of length and biomass of 10–15 mature specimens only. We defined mature individuals as those with the length of new blades exceeding the minimum size (75 cm) observed for fertile individuals. The GEM programme at Young Sound uses approximately the same size delimitation by including blades with a total length of new and old blades of at least 1.5 m.

At most sites, cross-sections of the blades were cut (at ¼, ½ and ¾ of the blade length) of five mature individuals, weighed and frozen for later determination of dry weight (dw), carbon (C), nitrogen (N) and phosphorus (P) content. The cross-sections were ca. 5 cm broad in the flat central part of the blades, gradually broader at the wavy extremities, and were sampled to represent the wet weight/dry weight ratio as well as the nutrient content of the entire blade. Moreover, five discs (3.44 cm in diameter) were cut of the central, thick tissue (at ½ of the blade length) of at least five individuals, using a cork borer, to determine the specific dry weight. Total blade area was estimated as blade length multiplied by mean width. Biomass was determined as wet weight and converted to dry weight (at 60 °C) based on wet weight/dry weight relationships of the 5 cm cross-sections. C and N content of the dried material was analysed using an elemental analyser (RoboPrep-C/N) in line with a mass spectrometer (Tracer mass, Europa Scientific, UK), while P content was analysed colourimetrically after acid digestion (Koroleff, 1983; Danish Standard DS291).

The data set on growth of *S. latissima* and *S. longicruris* from Greenland was supplemented with literature estimates quantified at other latitudes to compare our findings with growth estimates across the entire distribution range. Where annual growth was assessed based on consecutive blade-marking, we added all seasonal estimates to produce an annual estimate.

### Data analyses

We characterized the morphology and allometric relationships of the kelps through plots of size relationships and occurrence of sori (fertile tissue). The average size (±standard error) of the new blade of mature individuals, expressed as length, area and biomass, was calculated for each site and used as a measure of annual growth. The depth extension and annual growth of kelp were then analysed as functions of geographical position, duration of the open-water period with light and water temperature across the latitudinal gradient. Moreover, the annual growth of kelp was analysed as functions of the duration of the open-water period and water temperature over the decadal time series from Young Sound. In this time series analysis, kelp growth was related to the duration of the open-water period of the sampling year from ice break to sampling date, as well as to the duration of the current year's plus the preceding year's open-water period to evaluate the relative effect of the current and the previous year's open-water period on kelp growth. We chose to conduct separate analyses of the relationship between kelp performance and latitude, open-water period and temperature, respectively, as the independent variables are tightly coupled.

For all empirical relationships we identified, among a set of models, the one giving the best fit, i.e. the highest coefficient of determination (*R*^*2*^). The models were simple linear functions as well as a set of asymptotic functions (exponential, Gaussian and spheric) applicable for situations where the response shows an initial increase and subsequently stabilizes at higher threshold levels of the independent variable. These functions are characterized by a ‘sill’, i.e. the difference between the threshold level, where the initial increase in response reaches the asymptote, and the intercept with the *y*-axis (‘nugget’, for spheric and Gaussian functions), as well as a ‘range’ identifying the level of the independent variable resulting in the threshold. For Gaussian models, the effective range, defined as the value where 95% of the threshold is reached, is √3*range. Latitude ranges are quantified as deviations from 90°. For the estimation procedure start parameters for sill and range were determined from plots of the data. Our model selection procedure required that the range of the modelled variables should be within the range of the observed independent values, and we rejected models with negative sill. The variation of the mean estimates of depth limits differed markedly between sites and we therefore weighted each mean estimate by 1/SE^2^, where the SE is the standard error of the particular mean, with the implication that means with large standard error were given a lower weight. Statistical analyses were conducted using ‘Model’ and ‘GLM’ procedures in SAS version 9.2 (SAS institute, Cary, NC, USA).

## Results

### Sea ice cover, light and water temperature

**Fig. 2:**
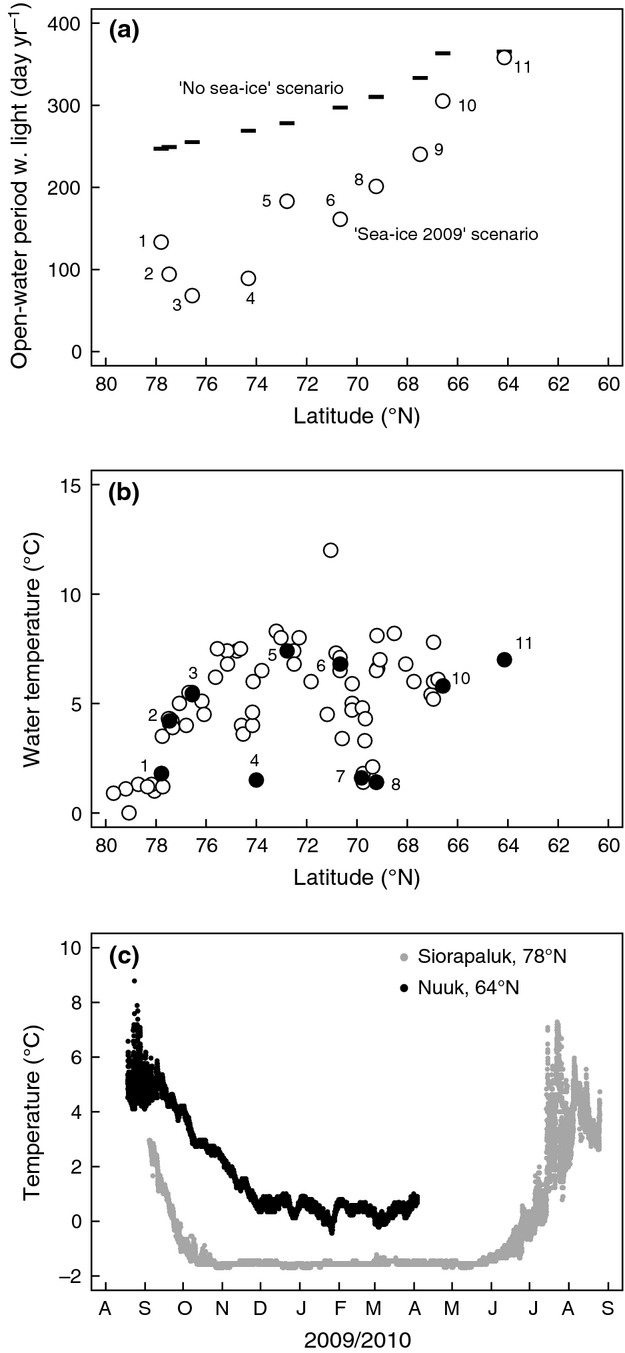
(a) Duration of the open-water period with light at the study sites along the Greenland coast in two scenarios: ‘Sea ice 2009’ (circles): representing combined information on the ice-free period in 2009 and the astronomical light period, and ‘No sea ice’ (slashes) representing the situation without sea ice. Study sites are numbered according to Table 1. Eqip Sermia (#7) lacks information on ice cover. (b) August water temperature at ca. 5 m depth along the Greenland coast, with filled symbols representing study sites. Equalunguit (#9) lacks information on temperature. (c) Seasonal changes in water temperature at 10 m water depth at the northernmost site (Siorapaluk, 78 °N) and the southernmost site (Nuuk, 64 °N).

At the northernmost site (78 °N) winter darkness lasted 118 days, leaving 247 days with light as opposed to an entire year with light below the polar circle (Fig. 2a). The length of the ice-free period ranged from 2 months (68 days) at Dundas (77 °N) to almost the entire year at Nuuk (64 °N) in 2009, and the combined effect of winter darkness and sea ice cover led to a wide range of the open-water period with light from 2 months in the north to almost an entire year in the south (Fig. 2a). Water clarity was quite similar at all five west coast sites at which it was measured between Ilulissat and Nuuk with light attenuation coefficients (K_d_) in August varying from 0.17 to 0.19 m^−1^ corresponding to Secchi depths of 13.9 m and 12.0 m, respectively, assuming 10% of surface irradiance at the Secchi depth (Højerslev, 1978). The water was clearer at Nuuk (K_d_ = 0.14 m^−1^; Secchi depth 16.4 m) and at Young Sound (K_d_ = 0.16 m^−1^, Secchi depth 14.4 m^−1^) (Table 1).

**Table 1:** Name and position of sampling sites, light attenuation (K_d_) measured near the sites and blade characteristics of the collected kelp, *Saccharina longicruris* or S. latissima^*^: specific dry weight (spec. dw) measured from weight vs. area relationships of the central, thick tissue and carbon (C, % of dw), nitrogen (N, % of dw), and phosphorus content (P, % of dw) of blade cross-sections. Data from August 2009 (tissue samples) and August 2010 (K_d_). Eqip Sermia and Ilulissat are sites with glaciers. Data are means ± standard error, the number of replicates (n) is 5, except at Eqalunguit (dw: *n* = 14), Nuuk (dw: *n* = 10, CN: *n* = 5; P: *n* = 3) and Young Sound (*n* = 15). For some of the sites, some variables are not determined (n.d.)

Site	Latitude (lat; lon)	K_d_m^−1^	Spec. dw mg dw cm^−2^	C % of dw	N % of dw	P % of dw
1. Siorapaluk	77°47′; 70°40′	0.18	n.d.	n.d.	n.d.	n.d.
2. Qaanaaq	77°28′; 69°15′	0.18	15.0 ± 2.6	36.85 ± 0.78	1.24 ± 0.10	0.22 ± 0.02
3. Dundas	76°33′; 68°52′	n.d.	14.9 ± 0.8	37.86 ± 0.41	0.77 ± 0.09	0.16 ± 0.02
4. Young Sound	74°19′; 20°14′	0.16	n.d.	37.96 ± 0.38	1.32 ± 0.07*	n.d.
5. Upernavik	72°47′; 56°10′	0.19	n.d.	n.d.	n.d.	n.d.
6. Uummannaq	70°40′; 51°36′	0.17	24.1 ± 4.0*	37.97 ± 0.73*	1.18 ± 0.10*	0.18 ± 0.02*
7. Eqip Sermia	69°45′; 50°21′	n.d.	12.6 ± 2.8*	33.05 ± 1.24*	3.16 ± 0.08*	0.46 ± 0.02*
8. Ilulissat	69°14′; 51°06′	0.19	n.d.	n.d.	n.d.	n.d.
9. Equalunguit	67°29′; 53°38′	n.d.	18.8 ± 0.9	n.d.	n.d.	n.d.
10. Itelleq	66°35′; 53°31′	n.d.	n.d.	n.d.	n.d.	n.d.
11. Nuuk (1)	64°08′; 51°37′	0.14	18.0 ± 1.1	35.95 ± 1.12	1.10 ± 0.09	0.18 ± 0.03
Nuuk (2)	64°08′; 51°35′		16.8 ± 0.9	35.31 ± 1.41	1.03 ± 0.09	0.15 ± 0.01

August surface water temperatures at the kelp sampling sites ranged from 1.4 °C (Eqip Sermia 70 °N) to 7.4 °C (Upernavik 73 °N), generally coldest in the north and near glaciers (Eqip Sermia and Ilulissat at 69–70 °N) (Fig. 2b). On an annual basis, kelps at the northernmost sites (exemplified by data from Siorapaluk) experienced persistently colder water and more constant winter temperatures than at the southernmost sites (Fig. 2c).

### Depth extension of kelp forests

The macroalgae formed a fringe along the shore. The intertidal zone, which had a vertical range from up to 5 m in Nuuk to 2.5 m in Qaanaaq, was often colonized by fucoid macroalgae, particularly along coast stretches protected from ice and strong waves, and with the highest abundance furthest south (Krause-Jensen and Marbà, pers. obs.). Kelps and other subtidal macroalgae took over below the intertidal zone, where they colonized the rocky sea floor as well as scattered stones on sandy bottom, forming extensive subtidal forests. Video surveys documented that kelp forests occurred along the entire investigated stretch of Greenland's coast. The kelps at these relatively protected sites were dominated by the species *Saccharina longicruris*, *S. latissima* and *Agarum clathratum*; and occasionally *Alaria esculenta,* which was primarily observed at Upernavik (Table S1), the more open of the investigated sites. These species were all identifiable on the videos as *S. longicruris* has characteristic long, hollow stipes lifting the base of the blades, while the blades of *S. latissima* generally lie more flat on the seafloor; *A. clathratum* has characteristic holes and *A. esculenta* has a distinct midrib. But the videos did not allow detailed assessment of species composition or relative abundance as the species often formed several layers with only the upper being visible. *A. clathratum* was observed at all sites on the west coast where it occurred intermixed with the other species and generally was the deepest occurring erect macroalga, with scattered tiny (cm-dm range) individuals often forming the depth limit. *S. latissima* was observed at all study sites except Itelleq and Nuuk (although it does occur elsewhere around Nuuk, pers obs.). *S. longicruris* was also observed at all sites except Uummannaq and Eqip Sermia (low image quality at this site prevented identification) (Table S1) and also in outer parts of Young Sound (pers. obs.). Filamentous brown algae, particularly *Desmarestia aculeata*, were commonly represented in the macroalgal forest, as were various red and green macroalgae (e.g. *Palmaria palmata, Ulva lactuca*), and the rocks were often covered by pink crusts of coralline macroalgae extending to the deepest water depths examined. Sea urchins (*Strongylocentrotus* sp.) were common on the seafloor among the algae, even at the northernmost sites (Table S1). This grazer sometimes occurred in large numbers where the algae were scarce, but as it was only visible on the video where the sea bottom was bare, it was not possible to relate algal cover to sea urchin density.

**Fig. 3:**
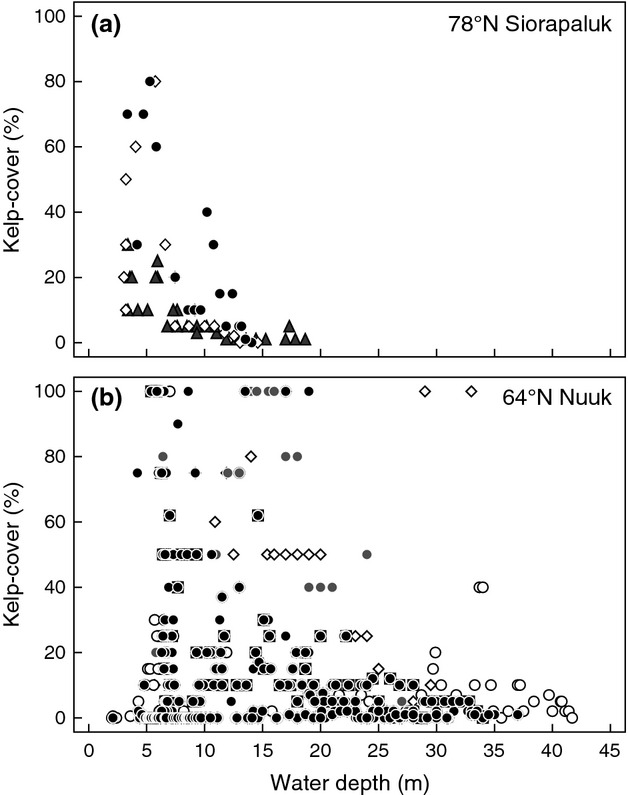
Cover vs. depth of kelp at 78 °N (Siorapaluk) and 64 °N (Nuuk) based on video surveys along 4–7 depth gradients/transect lines at each site, which are illustrated by different symbols.

**Fig. 4:**
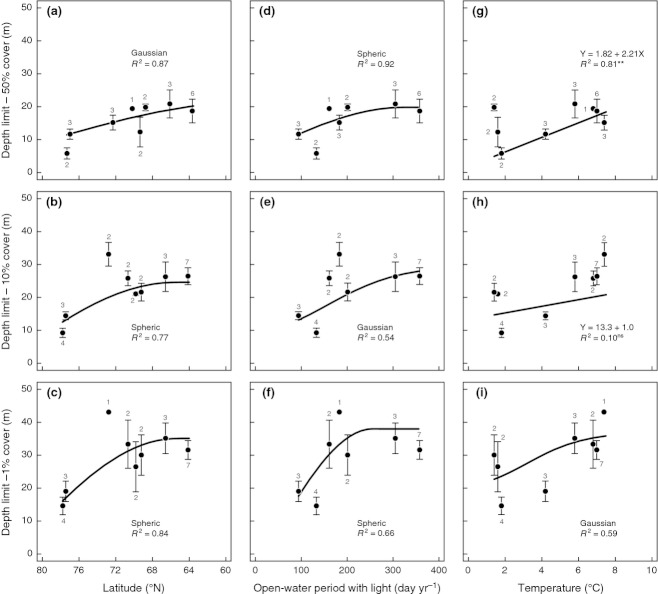
Depth limit of 50% (a, d, g), 10% (b, e, h) and 1% kelp cover (c, f, i) as functions of latitude (a–c), open-water period with light calculated by superimposing the ice-free period and the astronomical light period (d–f), and water temperature in August at 5 m (Nuuk and Young Sound ca. 10 m) depth (g–i). Depth limits represent means (±standard error) of the video-surveyed depth gradients from each site (*n* = 1–7, indicated for each site). The best fit of a set of tested models (linear Gaussian, spheric or exponential) is shown along with information on the coefficient of determination (*R*^*2*^) and for linear models also *P*-level (*P* > 0.05^ns^; *P* < 0.05*; *P* < 0.01**, *P* < 0.001). Further statistical details are given in Table S2. The number of data points varies slightly between panels because Eqip Sermia (#7) lacks information on ice cover, Equalunguit (#9) lacks information on temperature and a few sites did not have observations of 50% or 1% cover.

The cover of the kelp forest was highest near the shore and declined steeply towards deeper water (Fig. 3). The forest was narrow and shallow in the north, and broader, more abundant and deeper in the south (Fig. 3). Across the studied latitude gradient, the maximum depth of 50% kelp cover ranged from a minimum of 6 m to a maximum of 21 m, the depth of 10% kelp cover from 9 m to 33 m and the depth of 1% cover from 15 m to 43 m on average at the studied sites on Greenland's west coast (Fig. 4). The forest was shallow at the northernmost sites (Siorapaluk and Qaanaaq at 77–78 °N), but extended considerably deeper along the stretch of the coast from Upernavik at 73 °N to Nuuk at 64 °N. This latitudinal pattern was best described by asymptotic functions which explained 84–88% of the variation in depth limits (Fig. 4a–c; Table S2). The depth limit of 50% cover increased southwards until reaching a stable level of 23 m at a latitude of about 56 °N, while the depth limits of 10% and 1% cover reached stable levels of 24 m and 35 m at ∼65 °N (Fig. 4a–c; Table S2). Model parameters were connected with some uncertainty and not always significantly determined (Table S2).

The latitudinal pattern of the depth limit of the kelp forest was, to a large extent, related to large-scale increases in duration of the open-water period with light southwards (Fig. 4d–f, Table S2). The depth limit increased with the open-water period until a stable level was reached at an open-water period beyond 255 days. The open-water period explained 92% of the variation in the depth limit of 50% cover, 61% of the variation in depth limits of 10% cover and 66% of the variation in depth limits of 1% cover.

The depth limit of the kelp forest also related positively to the direct measurements of summer water temperature which explained 81%, 10% and 59% of the variation in the depth limit of 50% cover, 10% cover and 1% cover respectively (Fig. 4g–i; Table S2). The three study sites representing water temperatures below 2 °C exhibited a large variation in depth limits, which seemed related to differences in the duration of the open-water period; the kelp forest was confined to shallow waters at the two high-Arctic sites with 90-130 ice-free days, but extended considerably deeper at the similarly cold site near glaciers with ∼200 ice-free days (Ilulissat, 69 °N).

### Kelp growth along spatio-temporal gradients

**Fig. 5:**
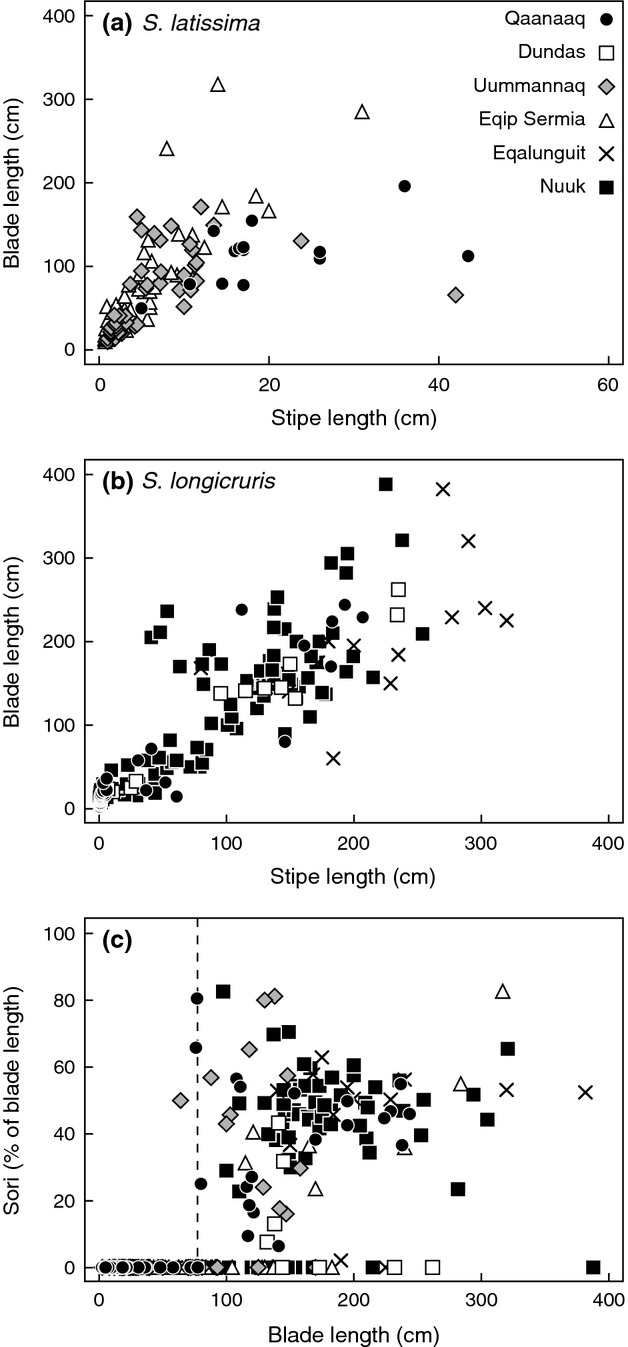
Morphometric relationship between blade length and stipe length of *S. latissima* (a) and *S. longicruris* (b), and between reproductive tissue and blade length of *S. latissima* and *S. longicruris* (c) from various sites along Greenland's west coast. The dashed line represents the minimum length of fertile individuals.

The size of the annual blade of *Saccharina,* which reflects the annual production of the kelp, increased as a function of the length of the perennial stipe for both the short-stiped *Saccharina latissima* (Fig. 5a) and the long-stiped *Saccharina longicruris* (Fig. 5b). Only individuals having blades larger than 75 cm were fertile, with sori developing in up to 80% of total blade length (Fig. 5c). The size of blades of such mature individuals (i.e. with blade length >75 cm) averaged 115–215 cm in length, 23–70 cm in width and 23–179 g dw in biomass among sites. Data from Qaanaaq on *S. longicruris* were omitted because only very few (seven) mature individuals were sampled and the demarcation between new and old blade was doubtful. Stipe lengths averaged 9–21 cm for mature *S. latissima* and 130–226 cm for *S. longicruris* across sites, and stipe diameter, which also increased as a saturating function of stipe length, reached an average of 4.6–8.9 mm for *S. latissima* and of 13.0–14.8 mm for *S. longicruris* across sites. The specific biomass of the central thick blade tissue varied from 12.6 to 24.1 mg dw cm^−2^ with the lowest value recorded at the glacier site, Eqip Sermia (70 °N), the highest at Uummannaq (70 °N), and intermediate levels further north and south (Table 1). Carbon content of the blades ranged from 33.05 to 37.97% of dw, nitrogen content from 0.77 to 3.16% of dw and phosphorus content from 0.15 to 0.46% of dw, with the lowest C levels and the highest nutrient levels observed near glaciers (Eqip Sermia) (Table 1).

**Fig. 6:**
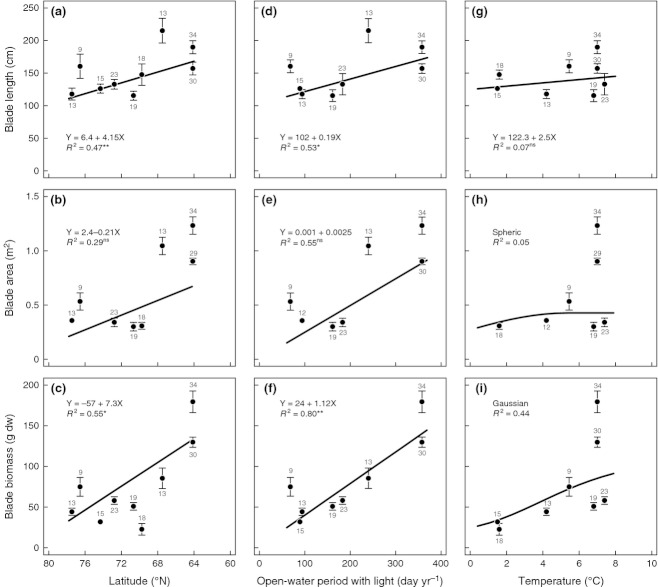
Annual production of *S. latissima* and *S. longicruris* expressed as length (a, d, g), area (b, e, h) and biomass (c, f, i) of the new blade of mature individuals as a function of latitude (a–c), open-water period with light (d–f), calculated by superimposing the ice-free period and the astronomical light period, and water temperature (g–i). Data points represent means (±standard error) and the number of observations is indicated for each site. The best fit of a set of tested models (linear Gaussian, spheric or exponential) is shown along with information on the coefficient of determination (*R*^*2*^) and for linear models, also *P*-level (*P* > 0.05^ns^; *P* < 0.05*; *P* < 0.01**, *P* < 0.001). Further statistical details are given in Table S3. The number of data points differs slightly between panels as Young Sound (74 °N) lacked information on blade width and thus blade area, Eqip Sermia (70 °N) lacked information on sea ice cover and Equalunguit (67 °N) lacked information on temperature.

Blade biomass ranged sevenfold, blade area fourfold, and blade length almost twofold across the latitudinal gradient. The mean blade size, and thus the annual production of mature individuals, generally increased from minimum levels in the north and near glaciers (at 69–70°N) to higher levels further south (Fig. 6a–c). The latitudinal pattern was best fitted by linear functions which explained 29–55% of the variation. The increase in production southwards was coupled to the longer open-water period; linear models explained 53–80% of the variation with the best fit obtained for production measured as biomass (Fig. 6d–f, Table S3). Water temperature alone was a less strong predictor explaining 5–44% of the variation (Fig. 6g–i; Table S3).

**Fig. 7:**
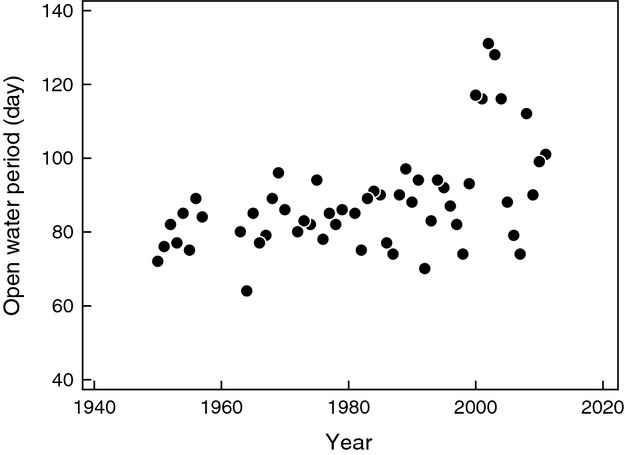
Open-water period 1950–2011 in Young Sound (74°N). Older data are extracted from the log book of the military patrol SIRIUS working in the area, whereas more recent data are from the Greenland Ecosystem Monitoring. Updated after Glud *et al*. (2007).

**Fig. 8:**
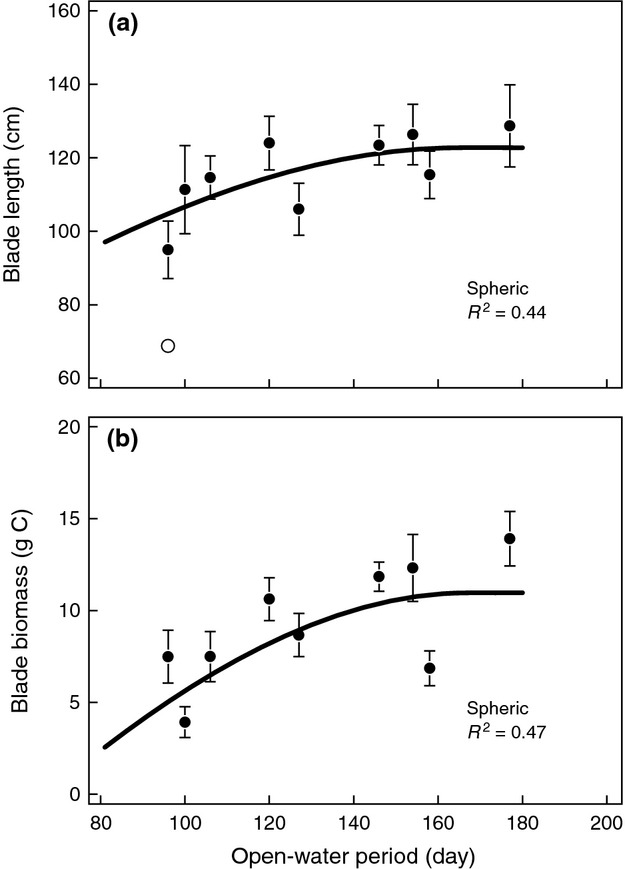
Size of the annual blade, measured as length (a) and biomass (b), representing the annual production of *S. latissima* in Young Sound, northeastern Greenland, as a function of the duration of the open-water period (of the preceding year and the current year until the day of sampling). Data represent means (± standard error) for the years 1999 (solely data on length growth, Borum *et al*., 2002) and 2003–2011. The best fit of a set of tested models (linear, spheric or exponential) is shown along with information on the coefficient of determination (*R*^*2*^) and for linear models also *P*-level (*P* > 0.05^ns^; *P* < 0.05*; *P* < 0.01**, *P* < 0.001). Further statistical details are given in Table S4.

In Young Sound, the duration of the open-water period was relatively constant from 1950 to 2000, but has generally been longer and more variable during the last decade (Fig. 7, updated from Glud *et al*., 2007). Since 1999, the duration of the ice-free period has varied by almost a factor two; length production of the kelp has shown a similar variation and kelp biomass production has varied by almost a factor four. Over this time period, up to 47% of the variation in kelp production could be explained by the duration of the open-water period (Fig. 8, Table S4). The explanatory power of the models increased considerably when not only the open-water period of the current year but also that of the preceding year was included. Water temperature alone only explained up to 31% of the variation in production.

## Discussion

### Drivers of kelp depth extension and production in Greenland

Our study indicates that large parts of Greenland's almost 90 000 km coastline are lined with kelp forests forming an extensive underwater forest composed of metre-long kelps in strong contrast to the tiny plants on land. The kelp forests penetrated deeper and were more productive in the south, paralleling large-scale increases in the duration of the open-water period and, thus, seasonal sea ice cover, which is an integrative variable strongly connected with water temperature. We suggest that this variable is a main driver of the latitudinal patterns, although empirical relationships do not necessarily imply causal effects. Seasonal sea ice cover explained up to 92% of the geographic variation in depth extension and 80% of the variation kelp production, while water temperature alone generally was a less strong predictor (Figs 4 and 6; Tables S2, S3). The time series from Young Sound confirmed and reinforced these findings.

The correlations between algal performance and the duration of the open-water period are surprisingly strong considering that the large-scale patterns in sea ice cover inferred from remote sensing cannot precisely describe ice conditions at the study sites and do not take into account that the light-blocking effect of sea ice varies depending on ice thickness, content of brine and air bubbles, as well as presence of snow and melt ponds (Glud & Rysgaard, 2007; Glud *et al*., 2007). The light climate at the sea bottom also depends strongly on water clarity. Our one-time measurements of light attenuation along the Greenland coast were insufficient for describing the strong seasonal variation in light attenuation e.g. related to the spring bloom of phytoplankton and inorganic particles from glacial and terrestrial run-off (Sejr *et al*., 2007; Jensen & Rasch, 2009) needed for estimating light attenuation on an annual basis. However, light attenuation was relatively similar across most sites and did not conflict with the identification of the open-water period as the main driver of kelp performance. The depth limit of kelps represents the compensation depth, where light energy for photosynthesis just balances carbon losses on an annual basis, except where intensive grazing or physical forces take over the control. A previous study in northeastern Greenland (Young Sound, 74 °N) estimated that 0.7–1.6% of total annual surface irradiance was available at the reported depth limit of 15–20 m, corresponding to 40–96 mol photons m^−2^ yr^−1^ (Borum *et al*., 2002) and similar to light levels at the lower depth limit found in other studies (50–70 mol photons m^−2^ yr^−1^) (Lüning & Dring, 1979; Chapman & Lindley, 1980; Dunton, 1990). Depth limits exhibited a marked initial response to longer open-water periods followed by stabilization at open-water periods beyond 255 days, indicating that the importance of this large-scale driver is indeed highest in the north and gradually levels off southwards, where other regulating factors, some of more local character, may play a larger role. As our latitudinal growth studies involved two species of *Saccharina* sampled at different sites, we cannot exclude a possible influence of species characteristics on the response patterns.

Seawater temperature also potentially exerts a major regulating effect on the kelp as all enzymatic processes related to plant metabolism are temperature-dependent (Brown *et al*., 2004; Gómez *et al*., 2009). The coarse measurements of summer temperature were a less strong predictor of algal performance than the open-water period, but both variables indicated a positive response of kelp performance to increasing water temperatures within the temperature range studied. This finding is line with the fact that most of the species, including *Saccharina latissima* and *S. longicruris,* are cold-temperate species many of which have temperature optima for growth well above ambient temperatures (Bischoff & Wiencke, 1993; Müller *et al*., 2009).

Seasonal nutrient limitation is likely a larger problem for planktonic algae than for perennial kelp which can buffer periodical nutrient shortage through tissue nutrient pools (Mann, 1973). Our sampling in late summer most probably represents annual minimum nutrient levels as stocks accumulated over the winter have been used for growth, and low levels are therefore not necessarily alarming. However, at Dundas (77 °N), tissue N-levels were very low (0.8% of dw) compared with the global average for macroalgae of 1.9% of dw (Duarte, 1992), and this population probably exists near the physiological limit of survival. Also at Qaanaaq, 77 °N, the apical half of *S. latissima* blades was entirely pale; and on the west coast in general, we did not observe individuals with intact old blades. The perennial life form with the old blade remaining active and translocating carbon and nutrients to new tissue (Mann, 1973), which is particularly well developed in the endemic Arctic *Laminaria solidungula* (Dunton, 1985; Dunton & Jodwalis, 1988), is also reported for *S. latissima* from Young Sound (Borum *et al*., 2002). By contrast, *S. latissima* from Alaska and Maine are apparently more poorly adapted to concurrent low temperature, low N and low light availability (Dunton, 1985; Korb & Gerard, 2000), and we speculate that the same may be the case for *S. latissima* in northwest Greenland, and that different ecotypes inhabit Greenland's west and east coast. In general, however, tissue nutrient concentrations did not indicate limitation as kelp production was lowest (23 g dw, Fig. 6) where tissue nutrient levels were highest (Eqip Sermia; 3.16% N and 0.46% P of dw, Table 1). The high nutrient concentration at Eqip Sermia is likely due to higher nutrient availability near areas of glacial discharge from entrainment of deeper nutrient-rich and saltier waters (Arendt *et al*., 2010; Mortensen *et al*., 2011). Low growth rates at this site probably reflect light limitation or low temperature.

Bottom structure, exposure and grazing are additional important regulating factors, and many of our observations of low or no algal cover along the depth gradients were associated with lack of hard bottom or high abundance of sea urchins. The very high depth penetration of kelp in Upernavik may further reflect that this site is relatively exposed, as suggested by the open location, which ensures good circulation and limits deposition of material on the blades. Sea urchins were frequent even at the northernmost sites, and often associated with a dominance of *Agarum clathratum*, which is relatively resistant towards grazing (Gagnon *et al*., 2005). A recent study in Disko Bay reported particularly deep macroalgal belts at exposed sites with rocky bottom and low density of grazers (Hansen JLS, Hjorth M, Rasmussen MB, Bruhn A, Christensen PB, unpublished), with depth limits exceeding those found at the relatively protected sites included in our study.

### Macroalgal production and performance across larger geographical ranges

**Fig. 9:**
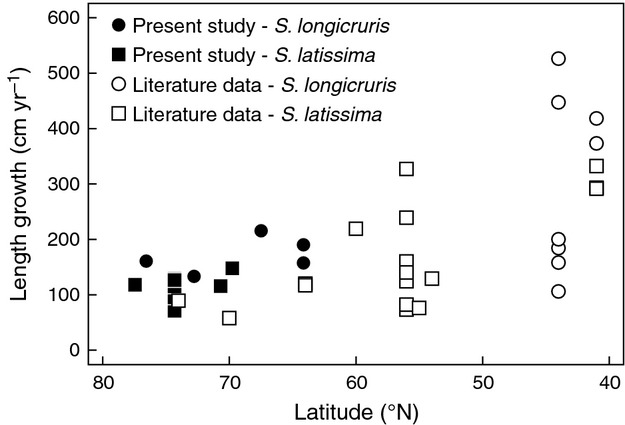
Annual length growth of *Saccharina latissima* and *S. longicruris* as functions of latitude across the entire geographical distribution range of the species. Data from the present study (filled symbols) were compared with data from the literature (open symbols) where annual growth was typically assessed based on consecutive blade-marking, in which case we added all seasonal estimates to produce an annual estimate. Studies included Parke, 1948; Mann, 1973; Weile, 1996; Chapman & Craige, 1977; Johnston *et al*., 1977; Brady-Campbell *et al*., 1984; Conolly & Drew, 1985; Dunton, 1985; Egan & Yarish, 1990; Sjötun, 1993; Sjötun & Gunnarson, 1995; Schaeffelke *et al*., 1996; Borum *et al*., 2002; Vadas *et al*., 2004; R. Thinggaard unpublished.

Although the mean annual kelp growth of 1.15–2.15 m for the study sites along the Greenland coast seems impressive, a synthesis of data from the literature covering the entire distribution range of *Saccharina* showed that the Greenland data fall in the lower range of the global data set, and that the upper boundaries of annual kelp production continue to increase southwards in the temperate zone (Fig. 9). The production of kelps from southern sites showed considerable variability, however, and only rarely reached the upper boundary, indicating that factors in addition to those governed by the large-scale latitudinal gradient played important regulating roles (Fig. 9). Even near the southern distribution limit of *S. latissima* at 40 °N, the production rate was potentially very high, suggesting that the lethal temperature is abrupt or that other phases of the life cycle and/or ecological interactions limit the expansion of the species further south. The few reports on temperature tolerance of various life stages of *S. latissima* are not conclusive on this matter but show relatively high temperature optima for production of the blade (10–15 °C) and an upper lethal temperature as high as 23 °C (Bolton & Lüning, 1982; Müller *et al*., 2009). There are also indications that thresholds of kelp resistance to additional stressors may decline with warming (Wernberg *et al*., 2010).

In accordance with the increase in depth limits and production of Greenland kelps southwards, studies from Antartica, which does not have kelps, also report higher macroalgal biomass and species richness with increasing distance from the pole (DeLaca & Lipps, 1976; Moe & DeLaca, 1976). At lower latitudes (10–60 °N), however, the highest levels of macroalgal biomass and diversity across depth ranges were found at 45–60 °N and in the low intertidal and high subtidal both variables declined southwards (Konar *et al*., 2010).

### Climate forcing responses along temporal and spatial gradients and predictions for the future

The Arctic region is characterized by a scarcity of time series data, not only with respect to kelp but also for marine biota and marine ecosystems in general (Wassmann *et al*., 2011). Response to climate forcing along spatial gradients is therefore an important supplement for predicting responses of the marine biota to climate change. In this study, macroalgal performance along spatial and temporal gradients show striking similarities, and together suggest that global climate change will increase the importance of kelp in a warmer Arctic with less sea ice. One of the few other time series on macroalgae from the Arctic is from the rocky intertidal of Svalbard, where macroalgal biomass has tripled, the algae have moved higher on the shore and the number of taxa has doubled between 1988 and 2008, paralleling increases in temperature and decreases in sea ice cover (Weslawski *et al*., 2010). North Atlantic polar to cold-temperate seaweeds are predicted to extend their distribution into the high-Arctic based on information on temperature tolerance combined with present and projected isotherms, but retreat along the northeastern Atlantic coastline over the 21st century (Müller *et al*., 2009). Intertidal biota are already moving northwards at speeds of up to 50 km per decade (Helmuth *et al*., 2006; Hawkins *et al*., 2008; Weslawski *et al*., 2010), but a recent review identified no evidence yet of changed distribution ranges of the dominant kelp along the northwest Atlantic shores of Canada (Merzouk & Johnson, 2011).

In summary, kelp forests form an extensive underwater forest along the Greenland coast, and our spatio-temporal studies suggest that this forest will expand in a warmer future as the length of the ice-free period increases. As kelps are foundation species that modify the environment and resource availability by supporting a rich biodiversity of associated flora and fauna and having profound effect on C-fluxes and secondary production, an expansion of kelp forests will exert cascading effects on the coastal Arctic ecosystem. Positive consequences may, however, be partly offset by the potential decline of endemic Arctic kelps.

## Supporting Information

**Table S1.** Overview of video transects with indication of site, transect (Tr.) number, position, water depth, coastal slope (coarsely assessed based on UTM coordinates and depth), presence of *Saccharina latissima* (S. lat), *Saccharina longicruris* (S. lon), *Agarum clathratum* (A. cla), *Alaria esculenta* (A. esc), sea urchins and visible bottom features.

**Table S2.** Latitudinal gradients: Empirical relationships between the response variables (Resp. var.) ‘depth limit of 50%, 10% and 1% kelp cover’ and the explanatory variables (Expl. var.) latitude, open-water period with light (Open w.) and water temperature (Temp.). Each relationship is fitted by the model (linear, exponential, Gaussian or spheric) giving the best fit (see plots in Fig. 4). Model parameters (Range, sill, nugget or intercept, slope), *P*-values and *R*^*2*^ are given. Latitude ranges are quantified as deviations from 90° and the effective range of Gaussian models is √3*range. Sill+nugget give the asymptote. All models are based on weighed data sets.

**Table S3.** Latitudinal gradients: Empirical relationships between the response variables (Resp. var.) ‘kelp production based on blade length (Prod.-length), blade area (Prod.-area) or blade biomass (Prod. −biom.) and the explanatory variables (Expl. var.) latitude, open-water period with light (Open w.) and water temperature (Temp.). Each relationship is fitted by the model (linear, exponential, Gaussian or spheric) giving the best fit (see plots in Fig. 6). Model parameters (Range, sill, nugget or intercept, slope), *P*-values and *R*^*2*^ are given. Latitude ranges are quantified as deviations from 90° and the effective range of Gaussian models is √3*range. Sill+nugget give the asymptote. All models are based on weighed data sets.

**Table S4.** Time-series analyses: Empirical relationships between the response variables (Resp. var.) ‘kelp production based on blade length (Prod.-length), or blade carbon biomass (Prod. – C. biom.) and the explanatory variables (Expl. var.) open-water period of the current year until sampling date (Open (1)), of the current year until sampling date and the preceding year (Open (1 + 2)) and water temperature (Temp.). Each relationship is fitted by the model (linear, exponential, Gaussian or spheric) giving the best fit (see plots in Fig. 8). Model parameters (Range, sill, nugget or intercept, slope), *P*-values and *R*^*2*^ are given and the effective range of Gaussian models is √3*range. Sill+nugget give the asymptote. All models are based on weighed data sets. Data from 1999 (Borum *et al*., 2002) are not included in the model as only kelp lengths were available.
