# Supplementary material for: Seasonal sea ice cover as principal driver of spatial and temporal variation in depth extension and annual production of kelp in Greenland
Source: Glob Chang Biol. 2012 Jul 26;18(10):2981–94. doi: 10.1111/j.1365-2486.2012.02765.x (PMC3597251; doi:10.1111/j.1365-2486.2012.02765.x)
Supplement: Supplementary file 1 [file gcb0018-2981-SD1.docx]

Table S1. Overview of video transects with indication of site, transect (Tr.) number, position, water depth, coastal slope (coarsely assessed based on UTM coordinates and depth), presence of *Saccharina latissima* (S. lat), *Saccharina longicruris* (S. lon), *Agarum clathratum* (A. cla), *Alaria esculenta* (A. esc), sea urchins and visible bottom features.

| **Date** | **Site** | **Tr.** | **Start position** | | **End position** | | **Slope** | ***S. lat*** | ***S. lon*** | ***A. cla*** | ***A. esc*** | **urchins** | **Visible bottom** |
| --- | --- | --- | --- | --- | --- | --- | --- | --- | --- | --- | --- | --- | --- |
| 30.08.09 | Uummannaq | 1 | 70 39.8249 | 51 35.9309 (3.5 m) | 70 39.7613 | 51 36.3769 (34 m) | 3.2º | x |  | x |  |  | sand, stones, rock |
| 30.08.09 | Uummannaq | 2 | 70 39.8118 | 51 36.6803 (2.8 m) | 70 39.7614 | 51 36.7394 (34 m) | 25.9 º | x |  | x |  | x | sand, stones, rock |
| 02.09.09 | Qaanaaq | 1 | 77 27.8708 | 69 15.9177 (3.6 m) | 77 27.7247 | 69 16.4308 (17.3 m) | 2.0 º | x | x | x |  | x | sand, stones, rock |
| 02.09.09 | Qaanaaq | 2 | 77 28.0200 | 69 15.7315 (4.3 m) | 77 27.7438 | 69 16.7846 (17.4 m) | 0.5 º | x | x | x |  | x | sand, stones, rock |
| 02.09.09 | Qaanaaq | 3 | 77 27.6347 | 69 13.0217 (4.9 m) | n.a. | n.a. |  | x | x | x |  | x | sand, stones, rock |
| 07.09.09 | Eqip Sermia | 1 | 69 45.5105 | 50 21.4695 (2.6 m) | 69 45.7152 | 50 22.1831 (25.5 m) | 1.9 º | x |  | x |  | x | sand, stones |
| 07.09.09 | Eqip Sermia | 2 | 69 45.6672 | 50 21.9031 (4.2 m) | 69 45.7577 | 50 22.1312 (27.5 m) | 2.8 º | x |  | x |  | x | sand, stones |
| 08.08.09 | Ilulissat | 1 | 69 14.2450 | 51 06.3895 (8.3 m) | 69 14.3019 | 51 06.4436 (29.1 m) | 12.7 º | x | x | x |  | x | rock, sand |
| 08.08.09 | Ilulissat | 2 | 69 14.3070 | 51 05.7697 (3.3 m) | 69 14.3937 | 51 05.9308 (32.1 m) | 2.6 º | x | x | x |  | x | rock, sand |
| 09.09.09 | Itelleq | 1 | 66 34:7558 | 53 30.6790 (3.5 m) | 66 34.6936 | 53 30.9223 (35.8 m) | 14.4 º |  |  | x |  |  | rock, shells |
| 09.09.09 | Itelleq | 2 | 66 34.4250 | 53 30.7276 (6.7 m) | 66 34.3833 | 53 30.8296 (34.7 m) | 24.4 º |  |  | x |  | x | rock, shells |
| 09.09.09 | Itelleq | 3 | 66 34.4989 | 53 30.6836 (4.0 m) | 66 34.4821 | 53 30.9631 (34.9 m) | 13.8 º |  | x | x |  | x | rock, shells |
| 04.09.09 | Siorapaluk | 1 | 77 47.0700 | 70 40.1946 (3.0 m) | 77 47.0056 | 70 40.2667 (14.6 m) | 9.1 º | x | x | x |  | x | sand, stones |
| 04.09.09 | Siorapaluk | 2 | 77 46,9092 | 70 40.9570 (3.3 m) | 77 46.9056 | 70 41.1996 (15.2 m) | 11.6 º | x | x | x |  | x | sand, stones |
| 04.09.09 | Siorapaluk | 3 | 77 47.1335 | 70 39.2195 (3.4 m) | 77 47.0929 | 70 39.2107 (18.7 m) | 17.0 º | x | x |  |  | x | sand, stones |
| 04.09.09 | Siorapaluk | 4 | 77 47.0793 | 70 38.7175 (5.1 m) | 77 47.0539 | 70 38.6582 (17.9) | 21.4 º | x | x |  |  | x | sand, stones |
| 29.08.10 | Upernavik | 1 | 72 47.4723 | 56 08.9874 (6.2 m) | 72 47.4013 | 56 09 2298 (20.3m) | 1.9 º | x | x | x | x | x | sand, stones, rock |
| 29.08.10 | Upernavik | 2 | 72 47.5922 | 56 09.0089 (1.8 m) | 72.47.6651 | 56 09.2964 (31.5 m) | 13.4 º | x | x | x | x | x | sand, stones, rock |
| 29.08.10 | Upernavik | 3 | 72 47.7957 | 56 08.2589 (3.5 m) | 72 48.0272 | 56 08 8223 (41.7 m) | 1.6 º | x | x | x | x | x | sand, stones, rock |
| 15.08.08 | Nuuk | 1 | 64 07.908 | 51 37.074 (6 m) | 64 07.947 | 51 37.979 (37 m) | 4.0 º |  | x | x | x | x | sand, stones, rock |
| 12.08.08 | Nuuk | 2 | 64 08.523 | 51 35.911 (5.8 m) | n.a. | n.a. (29 m) | gentle |  | x | x |  | x | sand, stones |
| 12.08.08 | Nuuk | 3 | 64 09 366 | 51 34 307 (5.7 m) | n.a. | n.a. (35 m) | gentle |  | x | x |  | x | sand, stones |
| 13.08.08 | Nuuk | 4 | 64 08 324 | 51 38 826 (4.8 m) | n.a. | n.a. (31 m) | steep |  | x | x |  | x | sand, stones, rock |
| 13.08.08 | Nuuk | 5 | 64 09.221 | 51 36.014 (5.8 m) | n.a. | n.a. (34 m) | steep |  | x | x |  | x | sand, stones, rock |
| 13.08.08 | Nuuk | 6 | 64 10 155 | 51 34.575 (5.7 m) | n.a. | n.a. (28.7 m) | steep |  | x | x |  | x | sand, stones, rock |
| 15.08.08 | Nuuk | 7 | 64 08.512 | 51 35 080 (2 m) | 64 08.383 | 51 35.536 (41.7 m) | gentle |  | x | x |  | x | sand, stones, rock |

Table S2. Latitudinal gradients: Empirical relationships between the response variables (Resp. var) ‘depth limit of 50%, 10% and 1% kelp cover’ and the explanatory variables (Expl. var.) latitude, open-water period with light (Open w.) and water temperature (Temp.). Each relationship is fitted by the model (linear, exponential, Gaussian or spheric) giving the best fit (see plots in Fig. 4). Model parameters (Range, sill, nugget or intercept, slope), p-values and R^2^ are given. Latitude ranges are quantified as deviations from 90º and the effective range of Gaussian models is √3*range. Sill+nugget give the asymptote. All models are based on weighed data sets.

| Resp. var. | Expl. var. | Best fit |  |  | Parameter | | | R^2^ |
| --- | --- | --- | --- | --- | --- | --- | --- | --- |
|  |  |  | Intercept  (p-value) | Slope  (p-value) | Range  (p-value) | Sill  (p-value) | Nugget  (p-value) |  |
| Depth limit  -50% cover | Latitude | Gaussian |  |  | 19.1  (0.301) | 17.2  (0.078) | 5.7  (0.428) | 0.865 |
|  | Open w. | Spheric |  |  | 321.7  (0.055) | 14.2  (0.041) | 5.6  (0.131) | 0.923 |
|  | Temp. | Linear | 1.815  (0.090) | 2.21  (0.006) |  |  |  | 0.810  (0.006) |
| Depth limit  -10% cover | Latitude | Spheric |  |  | 24.5  (0.028) | 38.4  (0.058) | -13.8 (0.468) | 0.773 |
|  | Open w. | Gaussian |  |  | 218.5  (0.288) | 19.5  (0.209) | 9.8  (0.156) | 0.538 |
|  | Temp. | Linear | 13.3  (0.031) | 1.00  (0.454) |  |  |  | 0.097  (0.454) |
| Depth limit  -1% cover | Latitude | Spheric |  |  | 24.6  (0.003) | 60  (0.039) | -24.9  (0.299) | 0.842 |
|  | Open w. | Spheric |  |  | 255.1  (0.001) | 43.2 (0.168) | -5.2  (0.815) | 0.657 |
|  | Temp. | Gaussian |  |  | 4.4  (0.414) | 15.5  (0.170) | 21.2  (0.031) | 0.588 |

Table S3. Latitudinal gradients: Empirical relationships between the response variables (Resp. var) ‘kelp production based on blade length (Prod.-length), blade area (Prod.-area) or blade biomass (Prod. –biom.) and the explanatory variables (Expl. var.) latitude, open-water period with light (Open w.) and water temperature (Temp.). Each relationship is fitted by the model (linear, exponential, Gaussian or spheric) giving the best fit (see plots in Fig. 6). Model parameters (Range, sill, nugget or intercept, slope), p-values and R^2^ are given. Latitude ranges are quantified as deviations from 90º and the effective range of Gaussian models is √3*range. Sill+nugget give the asymptote. All models are based on weighed data sets.

| Resp.var. | Expl var | Best fit | Parameter | | | | | R^2^ |
| --- | --- | --- | --- | --- | --- | --- | --- | --- |
|  |  |  | Intercept  (p-value) | Slope  (p-value) | Range  (p-value) | Sill  (p-value) | Nugget  (p-value) | (p-value) |
| Prod.  -length | Latitude | Linear | 60.4  (0.099) | 4.15  (0.041) |  |  |  | 0.472  (0.041) |
|  | Open w. | Linear | 102.4  (<0.001) | 0.19  (0.041) |  |  |  | 0.528  (0.041) |
|  | Temp. | Linear | 122.3  (0.001) | 2.53  (0.529) |  |  |  | 0.069  (0.529) |
| Prod.  - area | Latitude | Linear | 2.42  (0.178) | -0.21  (0.621) |  |  |  | 0.287  (0.178) |
|  | Open w. | Linear | 0.0012  (0.993) | 0.0025  (0.055) |  |  |  | 0.553  (0.055) |
|  | Temp. | Spheric |  |  | 6.32  (0.447) | 0.21  (0.736) | 0.217  (0.675) | 0.046 |
| Prod.  - dw biom | Latitude | Linear | -57  (0.303) | 7.31  (0.022) |  |  |  | 0.553  (0.022) |
|  | Open w. | Linear | 24  (0.003) | 1.12  (0.932) |  |  |  | 0.800  (0.003) |
|  | Temp. | Gaussian |  |  | 6.91  (0.648) | 83.94  (0.677) | 22.76  (0.326) | 0.435 |

Table S4. Time-series analyses: Empirical relationships between the response variables (Resp. var) ‘kelp production based on blade length (Prod.-length), or blade carbon biomass (Prod. –C. biom.) and the explanatory variables (Expl. var.) open-water period of the current year until sampling date (Open (1)), of the current year until sampling date and the preceeding year (Open (1+2)) and water temperature (Temp.). Each relationship is fitted by the model (linear, exponential, Gaussian or spheric) giving the best fit (see plots in Fig. 8). Model parameters (Range, sill, nugget or intercept, slope), p-values and R^2^ are given and the effective range of Gaussian models is √3*range. Sill+nugget give the asymptote. All models are based on weighed data sets. Data from 1999 (Borum et al. 2002) are not included in the model as only kelp lengths were available.

| Resp. var. | Expl. var. | Best fit |  |  | Parameter | | | R^2^ |
| --- | --- | --- | --- | --- | --- | --- | --- | --- |
|  |  |  | Intercept  (p-value) | Slope  (p-value) | Range  (p-value) | Sill  (p-value) | Nugget  (p-value) | (p-value) |
| Prod.  -length | Open (1) | Linear | 90.08  (0.0006) | 0.252  (0.123) |  |  |  | 0.305  (0.122) |
|  | Open (1+2) | Spheric |  |  | 164.8  (0.015) | 79.7  (0.0.260) | 43.0  (0.539) | 0.444 |
|  | Temp. | Gaussian |  |  | 1.29  (0.685) | 11.7  (0.463) | 111.2  (<0.001) | 0.197 |
| Prod.  - C biom | Open (1) | Spheric |  |  | 130.6  (0.102) | 21.3  (0.526) | -10.9  (0.750) | 0273 |
|  | Open (1+2) | Spheric |  |  | 166.0  (0.014) | 25.2  (0.234) | -14.3  (0.495) | 0.472 |
|  | Temp. | Linear | 8.45  (0.001) | 2.13  (0.230) |  |  |  | 0.198  (0.230) |
